# Renal Function in Hypertensive Patients Receiving Cilnidipine and L-Type Calcium Channel Blockers: A Meta-Analysis of Randomized Controlled and Retrospective Studies

**DOI:** 10.7759/cureus.27847

**Published:** 2022-08-10

**Authors:** Mayakalyani Srivathsan, Vikram Vardhan, Azra Naseem, Sayali Patil, Vivek Rai, Deepakkumar G Langade

**Affiliations:** 1 Pharmacology, DY Patil University School of Medicine, Navi Mumbai, IND; 2 Anaesthesia, DY Patil University School of Medicine, Navi Mumbai, IND

**Keywords:** meta-analysis, renal function, hypertensive, l-type ccbs, cilnidipine

## Abstract

Nearly 65%-95% of chronic kidney disease (CKD) patients have hypertension. Calcium-channel blockers are the first-line drugs for the treatment of hypertension, including hypertension with diabetes. This study aims to estimate the effect of an L-type calcium channel blocker (CCB), cilnidipine, on the renal function of hypertensive patients. Randomized control trials were selected from PubMed, Embase, Google Scholar, China National Knowledge Infrastructure (CNKI), Science Direct, Elton B. Stephens Company (EBSCO), Springer, Ovid, Cochrane Library, Medline, VIP, and Wanfang databases (from the date of databases' establishment till January 2022). Data were independently evaluated following the Cochrane risk-of-bias tool. The changes in serum creatinine (SCr), urinary protein excretion (UPE), urinary protein/creatinine ratio (UPCR), and estimated glomerular filtration rate (eGFR) before and after treatment, in percentages, were extracted for the meta-analysis. The mean difference (MD) and a CI of 95% were determined using RevMan 5.3 software. A total of 11 studies were analyzed. The standardized mean difference (SMD) between cilnidipine and L-type CCBs was -0.022, suggesting a reduced SCr with cilnidipine. For UPCR, the SMD value is 1.28. Although cilnidipine reduced UPCR in all four studies, the L-type CCBs reported a slight increase in UPCR. For eGFR, the SMD value was found to be 0.693. Cilnidipine had a more favorable effect on eGFR when compared to the L-type CCBs. While cilnidipine had similar effects on SCr to that of L-type CCBs, cilnidipine showed greater improvement in UPCR, UPE, and eGFR values.

## Introduction and background

Hypertension is a continuous and genuine threat to public health. Most patients with chronic kidney disease (CKD) also suffer from hypertension, showing a close association. Hypertension is a risk factor for deterioration of kidney function, resulting in end-stage renal disease (ESRD) [1]. CKD, in return, has an adverse effect on blood pressure and may contribute to refractory hypertension. Both these illnesses can increase morbidity and mortality [2]. On the other hand, the improvement in renal function can help reduce the incidence of cardiovascular events.

Calcium-channel blockers (CCBs) were considered first-line treatment for hypertension, including in patients with diabetes. CCBs being potent vasodilators, are particularly effective in reducing peripheral resistance. Dihydropyridines like amlodipine, nifedipine, and felodipine are currently the most frequently used CCBs. Cilnidipine is a derivative of dihydropyridines and possesses the property of blocking both L-type and N-type calcium channels that are present on peripheral nerve endings inhibiting calcium release in sympathetic nerves and release of norepinephrine (NE). Sympathetic stimulation in the renal afferent and efferent arterioles increases resistance and decreases renal blood flow. Renal ischemia will cause further sympathetic activation, releasing pressor substances, such as NE and renin, leading to aggravated hypertension. Therefore, hypertension and CKD can cause mutual deterioration, and the therapeutic outcomes of hypertension are closely related to renal function.

Ca^2+^ channels are classified into six subtypes: L-, N-, P-, Q-, R-, and T- [3]. Of these, notable is the L-type, predominantly present in the heart and blood vessels, and the N-type present in the sympathetic nerve endings. The N-type calcium channels regulate the release of noradrenaline (NA), the neurotransmitter for the sympathetic system. Subsequently, the sympathetic system controls renal hemodynamics via alpha-adrenoceptor and glomerular filtration rate (GFR). In addition, the release of renin from the juxtaglomerular apparatus is incumbent upon the stimulation of the beta-1 adrenoreceptor.

L-type CCBs are used prolifically as anti-hypertensive agents. Nifedipine, the first CCB to be introduced in the 1960s, had a quick onset vasodilatory action. Disadvantageously, the rapid fall in blood pressure led to an excessive increase in heart rate as the body's sympathetic system tried to compensate. The disconcertment of reflex tachycardia steered research into better alternatives.

The second-generation CCBs, namely benidipine, efonidipine, manidipine, and nitrendipine, have a slower vasodilatory action, thus reducing the sympathetic reflex. Third-generation CCBs like amlodipine and azelnidipine further slow down the process and have minimal heart rate changes.

Cilnidipine has cemented its position as a unique CCB by having dual action of L-type and N-type Ca^2+^ Channels. As mentioned earlier, N-type Ca^2+^ channels play a vital role in neurotransmitter release from sympathetic nerve endings, and blocking it may suppress the overactivity of the cardiac sympathetic system [4].

These findings of a meta-analysis of all the published studies that met our inclusion criteria were conducted to evaluate the effect of cilnidipine and L-type CCBs on renal function. We also evaluated the results to provide evidence-based medical references for clinical treatments.

## Review

Material and methods

The systematic review has been performed according to the guidelines provided by the Preferred Reporting Items for Systematic Reviews and Meta-Analysis (PRISMA).

Study Selection

Studies meeting the following criteria were included as part of the analysis: (1) Purpose of the study was to evaluate the effects of cilnidipine and L-type CCBs on renal function in patients with hypertension; (2) the study design was either a prospective RCT or a retrospective cohort study; (3) Tests for urinary protein excretion (UPE), urinary protein/creatinine ratio (UPCR), serum creatinine (SCr), and the estimated glomerular filtration rate (eGFR) levels were performed before and after the treatment; and (4) Long-term treatment (12 weeks or more) with cilnidipine or L-CCBs was carried out.

Studies excluded from our analysis were (1) the studies where the renal function was not discussed; and (2) studies lacking adequate data on pre/ post treatment UPE, UPCR, SCr, and eGFR levels.

Data Source and Retrieval

Research articles from databases such as PubMed, Embase, Google Scholar, China National Knowledge Infrastructure (CNKI), Science Direct, Elton B. Stephens Company (EBSCO), Springer, Ovid, Cochrane Library, Medline, VIP, and Wanfang, within the period from the date of databases' establishment to January 2022 were searched. Keywords in English included "cilnidipine", "calcium channel blocker", "hypertension", "renal function", "urinary/urine protein excretion", "urinary/urine protein to creatinine ratio", "proteinuria", "randomly" and so on. Publications were not limited to any date or language. The relevant studies were then identified by a manual search of secondary sources, including references of initially identified articles as well as reviews and commentaries. All references were downloaded and then consolidated. They were checked for duplicates, after which further analyses were carried out.

Data Extraction and Quality Assessment

Two independent investigators evaluated the eligible articles. In case of a difference of opinions between the two investigators, a third investigator was invited, and the disputes were settled through discussion. The indices included SCr, UPE, UPCR, and eGFR. Basic data about eligible RCTs, sample capacity, features of the subject, intervention measures, observation period, and results were extracted from each individual study.

The articles were evaluated for risk of bias according to the Revised Cochrane risk-of-bias tool for randomized trials (RoB 2), which has five domains: Domain 1: Risk of bias arising from the randomization process. Domain 2: Risk of bias due to deviations from the intended interventions (effect of assignment to intervention). Domain 3: Risk of bias due to missing outcome data. Domain 4: Risk of bias in the measurement of the outcome. Domain 5: Risk of bias in the selection of the reported result. Each domain has signaling questions with designated responses of either Yes/Probably Yes/Probably No/No or No Information.

Statistical analysis

For each study, percentage changes in SCr, UPE, UPCR, and eGFR levels from the pre-treatment to the post-treatment in both the cilnidipine and L-type CCB groups were used to generate mean differences (MDs) and 95% CIs. If the SDs were unavailable, but the pre-/post-treatment SCr, UPE, UPCR, and eGFR levels were reported, missing SDs were recorded, and the changes in SCr, UPE, UPCR, and eGFR levels from pre-treatment to post-treatment were obtained according to the Cochrane Handbook. All the analyses were conducted using Review Manager version 5.3 (Nordic Cochrane Centre, Copenhagen, Denmark).

Heterogeneity analysis

The heterogeneity of the data was analyzed by calculating the Q-value and I^2^. In a value >50%, which suggested that heterogeneity existed among studies, the random-effect model was used for meta-analysis.

Bias analysis

Egger's test and funnel plot were used to identify whether the publication bias existed.

Results

**Figure 1:**
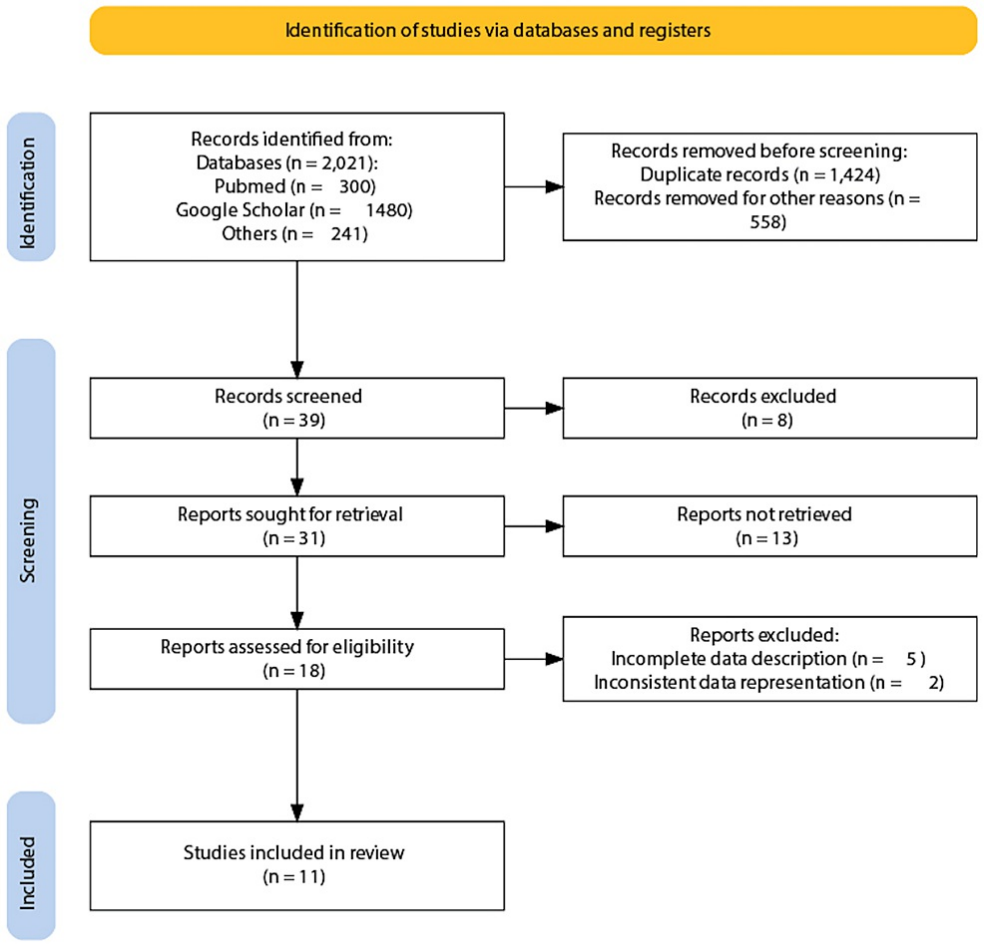
PRISMA statement flowchart on the literature search and study selection process. PRISMA: Preferred Reporting Items for Systematic Reviews and Meta-Analyses.

In the present investigation, a total of 2021 relevant studies were retrieved, and 1982 of them were excluded due to duplication, non-RCT research, absence of control group, absence of coincident purpose, or the form of a case report. Among the remaining 39 articles, 28 were further removed during the screening process. Finally, 10 RCTs [5-14] and one retrospective study [15] were selected for meta-analysis. All selected studies treated patients with cilnidipine or L-type CCBs (amlodipine, nifedipine, and others) for 12 weeks to 24 months. Figure 1 illustrates the literature selection process according to the PRISMA guidelines.

Table 1 shows the basic information of the selected trials.

**Table 1:** Basic information of the selected trials. CKD: Chronic kidney disease; eGFR: Estimated glomerular filtration rate; SCr: Serum creatinine; UPCR: Urine protein creatinine ratio; UPE: Urine protein excretion.

Trial	Inclusion Criteria	Duration	Intervention	Control	Number of patients Cilnidipine (L-type CCB)		Outcomes
Kojima S et al. (2004) [5]	Hypertension	12 months	Cilnidipine (10 mg/day)	Amlodipine (5 mg/day)	14	14	SCr, UPCR
Fujita T et al. (2007) [6]	Hypertension	12months	Cilnidipine (11.5± mg/day)	Amlodipine (5.3±2.4 mg/day)	147	130	SCr, UPCR
Morimoto S et al. (2007) [7]	Essential hypertension without renal disorders	24 weeks	Cilnidipine (10 mg/day)	Amlodipine (5 mg/day)	25	25	UPE
Satomura A et al. (2009) [8]	Hypertension with non-diabetic and chronic renal failure	12 months	Cilnidipine (N/A)	Amlodipine (N/A)	17	16	UPE
Konoshita T et al. (2010) [9]	Essential hypertension	12 weeks	Cilnidipine (10-20 mg/day)	Amlodipine (5-10 mg/day)	110	110	SCr, eGFR
Miwa Y et al. (2010) [10]	Hypertension with proteinuria >0.1 g/day	48 weeks	Cilnidipine (5-15 mg/day)	Amlodipine (2.5-5 mg/day)	18	17	UPE
Cao BQ et al. (2010) [11]	Essential hypertension with type-2 diabetes and renal damage	1 year	Cilnidipine (5-10 mg/day)	Felodipine (5-10 mg/day)	17	14	UPE
Ando K et al. (2013) [12]	Hypertension with type-2 diabetes and microalbuminuria	12 months	Cilnidipine (5-20 mg/day)	Amlodipine (2.5-10 mg/day)	163	167	SCr, eGFR
Abe M et al. (2013) [13]	Hypertension with stage 2-3 CKD	48 weeks	Cilnidipine (10-20 mg/day)	Amlodipine (2.5-5 mg/day)	35	35	SCr, eGFR
Kanaoka T et al. (2013) [14]	Hypertension with CKD	24 weeks	Cilnidipine (14.4±6.1 mg/day)	Amlodipine (N/A)	21	24	SCr, UPCR, eGFR
Oh MR et al. (2020) [15]	Hypertension with CKD	12 months	Cilnidipine/Efonidipine (N/A)	N/A	53	0	SCr, UPCR

Literature quality

The literature quality analysis was done using the criteria list provided by Cochrane. The literature was assessed on the basis of the method of randomization, blinding techniques, similarity in the groups at baseline, avoidance of cointerventions, acceptability among patients, dropout rate, the timing of the outcome assessment, and if the intention-to-treat analysis was included.

Of the 11 selected studies, not all described the method of randomization in detail, and most of the studies were open-label due to operational constraints. Adequate compliance to therapy was reported in all studies. The participant groups were similar at baseline. Intention-to-treat analysis was found to be followed only in one literature. Out of the 11 criteria to be checked, most of the studies were found compliant with 6-7 of them.

Heterogeneity analysis

We divided the data from all the studies into the SCr, UPCR, and eGFR groups for the analyses. Then, we calculated the Q-value and I^2^ for analyzing heterogeneity. The detailed heterogeneity information of these four groups has been described in Table 2. The analysis suggested that data from the SCr group had homogeneity while the data from the UPCR group had heterogeneity.

**Table 2:** Heterogeneity analysis. d.f.: Degree of freedom; eGFR: Estimated glomerular filtration rate; I^2^: Higgin's I-square value; Q-value: Cochrane's Q value; UPCR: Urine protein creatinine ratio.

Criteria	I^2^		d.f.	Significance level	Q-value
Serum creatinine (mg%)		0.00%	6	0.9928	0.7724
UPCR (g/gCr)		91.21%	2	0.1140	22.7641
eGFR (ml/min)		97.10%	3	<1.000	103.5585

Effect analysis

Effect on Serum Creatinine

Table 3 shows that seven studies (six RCT and one retrospective study) reported the changes in the SCr from pre-to post-treatment with cilnidipine (n=528) or L-type CCBs (n=516). Heterogeneity was detected in the data from the SCr group, and therefore we used the random effect model for further analysis.

**Table 3:** Changes in the SCr from pre-to post-treatment with cilnidipine or L-type CCBs. CCBs: Calcium channel blockers; SCr: Serum creatinine.

		Cilnidipine			L-type CCB		
Study	Duration (weeks)	Baseline mean (SD)	Post-treatment mean (SD)	Change mean (SD)	Baseline mean (SD)	Post-treatment mean (SD)	Change mean (SD)
Abe M et al. (2013) [13]	48	1.10 (0.10)	1.10 (0.10)	0.00 (0.10)	1.20 (0.10)	1.20 (0.10)	0.00 (0.10)
Ando K et al. (2013) [12]	48	0.77 (0.18)	0.79 (0.22)	0.02 (0.20)	0.78 (0.21)	0.81 (0.24)	0.03 (0.03)
Fujita T et al. (2007) [6]	48	1.27 (0.18)	1.37 (0.72)	0.10 (0.66)	1.29 (0.60)	1.45 (0.83)	0.16 (0.74)
Kanaoka T et al. (2013) [14]	24	2.00 (1.20)	2.10 (1.60)	0.20 (0.60)	1.80 (1.00)	2.00 (1.30)	0.10 (0.50)
Kojima S et al. (2004) [5]	48	1.36 (0.20)	1.50 (0.23)	0.14 (0.22)	1.11 (0.16)	1.14 (0.18)	0.14 (0.17)
Konoshita T et al. (2010) [9]	12	0.77 (0.32)	0.74 (0.25)	-0.03 (0.29)	0.77 (0.32)	0.75 (0.37)	-0.02 (0.35)
Oh MR et al. (2020) [15]	48	2.70 (0.43)	3.80 (3.8)	1.10 (0.48)	2.70 (0.43)	3.80 (0.49)	1.10 (0.48)

The standardized mean difference (SMD) between cilnidipine and L-type CCBs was -0.022 (95% CI: -0.143 to 0.0987), suggesting a greater but non-significant (p=0.719) reduction in SCr with cilnidipine.

There was minimal inconsistency in the data (I^2^=0.00%; 95% CI: 0.00 to 28.45; p=0.878).

Such results indicated no significant difference in terms of SCr between the two groups. Cilnidipine and L-type CCBs had similar effects on SCr in hypertensive patients.

**Figure 2:**
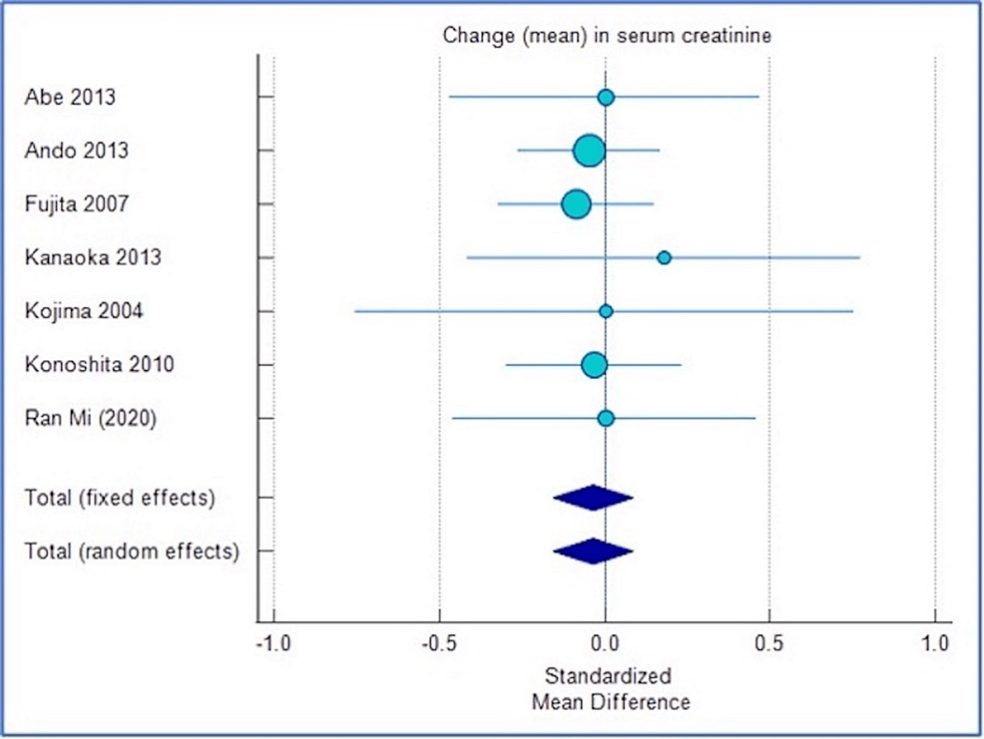
Forest plot showing change in SCr. The forest plot uses an effect measure of standardized mean difference [5,6,9,12,13,14,15]. The size of the bubble indicates the extent of SMD and the error bars represent 95% CIs for SMD. SCr: Serum creatinine; SMD: Standardized mean difference.

**Figure 3:**
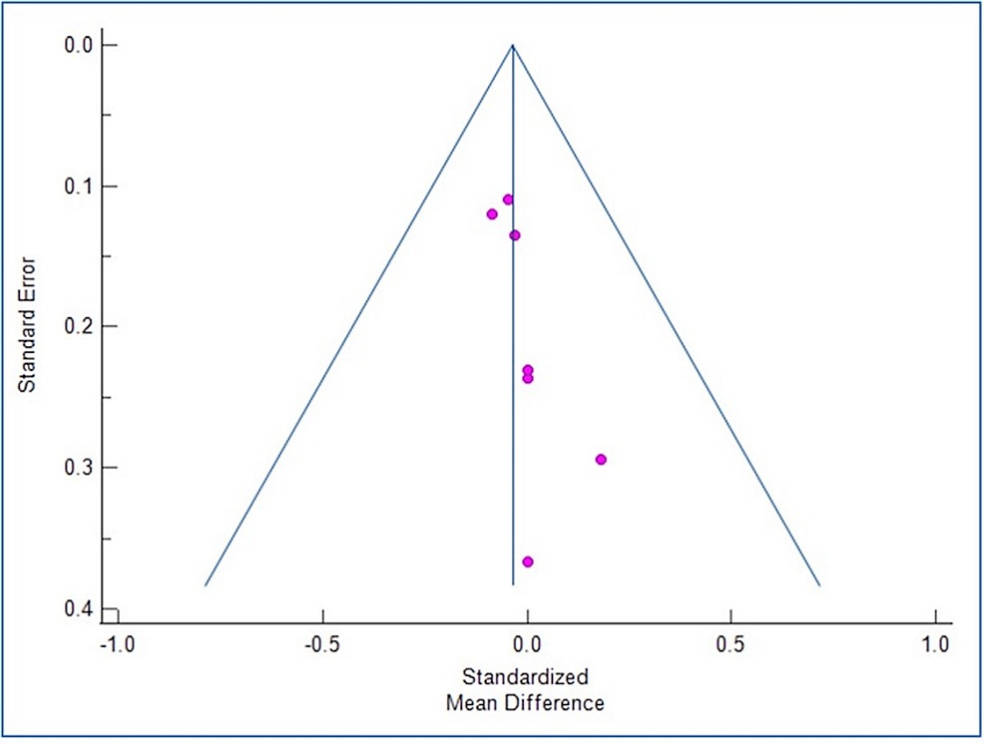
Funnel plot showing change in SCr. SCr: Serum creatinine.

Figures 2-3 show the corresponding forest plot and funnel plot showing the change in SCr. 

Effects on UPE

The results on the difference in UPE show a significant decrease in the values in the cilnidipine group as compared to amlodipine. The change was more in patients with CKD who already had a high protein loss in their urine [7,8,10,11]. Due to the lack of more studies and differences in the baseline characteristics of the participants taken for each study, we could not perform further statistical analysis.

Effects on UPCR

Table 4 shows that three RCTs reported the changes in UPCR from pre- to post-treatment with cilnidipine (n = 182) and L-type CCBs (n = 165). In the UPCR group, data heterogeneity was detected, and therefore we used a random-effect model for further analysis.

**Table 4:** Changes in the UPCR from pre-to post-treatment with cilnidipine or L-type CCBs. CCBs: Calcium channel blockers; UPCR: Urine protein-creatinine ratio.

		Cilnidipine			L-type CCB		
Study	Duration (weeks)	Baseline mean (SD)	Post-treatment mean (SD)	Change mean (SD)	Baseline mean (SD)	Post-treatment mean (SD)	Change mean (SD)
Fujita T et al. (2007) [6]	48	1.92 (2.13)	1.31 (0.12)	-0.61 (1.51)	1.71 (1.57)	1.88 (0.19)	0.17 (1.12)
Kanaoka T et al. (2013) [14]	26	1.4 (2.6)	1.10 (1.8)	-0.30 (1.10)	1.00 (1.20)	1.10 (1.40)	0.10 (0.90)
Kojima S et al. (2004) [5]	48	0.93 (0.23)	0.82 (0.20)	-0.11 (0.12)	0.86 (0.21)	1.47 (0.44)	0.61 (0.27)

SMD value is 1.28, I^2^ = 91.21% (95% CI = 77.27, 96.60). These results indicated a significant difference in terms of UPCR between the two groups. While cilnidipine reduced UPCR in all four studies, the control groups reported a slight increase in UPCR.

**Figure 4:**
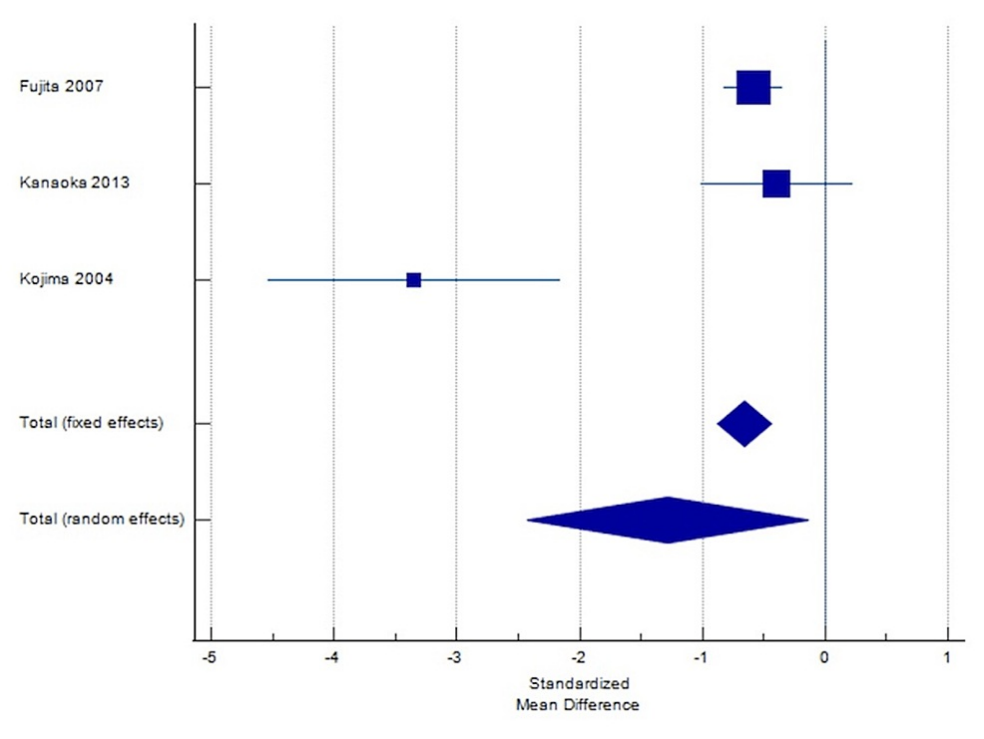
Forest plot showing change in UPCR. The forest plot uses an effect measure of standardized mean difference [5,6,14]. The size of the square indicates the extent of SMD, and the error bars represent the 95% CIs for SMD. SMD: Standardized mean differences; UPCR: Urine protein-creatinine ratio.

**Figure 5:**
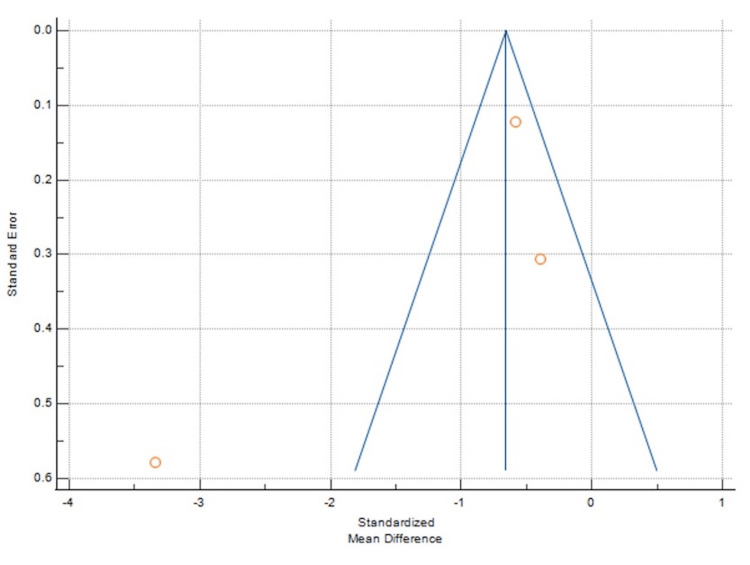
Funnel plot showing change in UPCR. UPCR: Urine protein-creatinine ratio.

Figures 4-5 show the corresponding Forest plot and Funnel plot showing the change in UPCR.

Effect on eGFR

Table 5 shows that four RCTs reported the changes in eGFR from pre- to post-treatment with cilnidipine (n = 329) and L-type CCBs (n = 336). In the eGFR group, data heterogeneity was detected, and therefore we used a random-effect model for further analysis.

**Table 5:** Changes in the eGFR from pre-to post-treatment with cilnidipine or L-type CCBs. CCBs: Calcium channel blockers; eGFR: Estimated glomerular filtration rate.

		Cilnidipine			L-type CCB		
Study	Duration (weeks)	Baseline mean (SD)	Post-treatment mean (SD)	Change mean (SD)	Baseline mean (SD)	Post-treatment mean (SD)	Change mean (SD
Abe M et al. (2013) [13]	48	48.00 (2.00)	49.00 (2.00)	1.00 (2.00)	46.00 (2.00)	46.00 (2.00)	0.00 (0.00)
Ando K et al. (2013) [12]	48	71.85 (15.85)	71.07 (17.96)	-0.78 (1.10)	73.48 (19.46)	70.89 (20.56)	-2.59 (0.90)
Kanaoka T et al. (2013) [14]	48	35.10 (17.30)	35.00 (19.00)	-0.10 (18.17)	34.90 (17.30)	34.10 (19.30)	-0.80 (18.33)
Konoshita T et al. (2010) [9]	12	71.50 (17.50)	73.70 (18.90)	2.20 (18.21)	71.50 (17.50)	74.20 (19.70)	2.70 (18.63)

SMD value was found to be 0.693, I2 =97.10% (95% CI = 77.27, 94.87, 98.36). These results indicated a significant difference in terms of eGFR between the two groups. Three out of four studies reported that the control group either showed no change or a decrease in the eGFR. In contrast, the cilnidipine group showed increased eGFR values in two studies. In all four studies, cilnidipine had a more favorable effect on eGFR when compared to the control drug.

**Figure 6:**
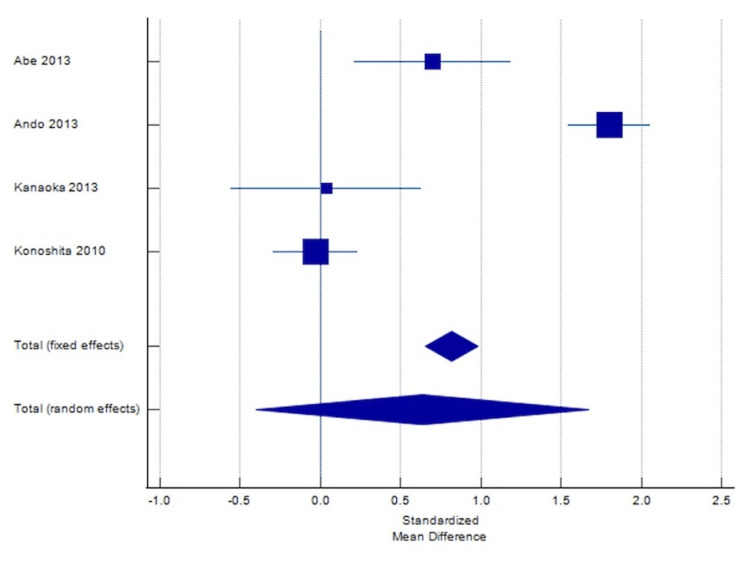
Forest plot showing change in eGFR. The forest plot uses an effect measure of standardized mean difference [9,12,13,14]. The size of the square indicates the extent of SMD, and the error bars represent the 95% CIs for SMD. SMD: Standardized mean differences; eGFR: Estimated glomerular filtration rate.

**Figure 7:**
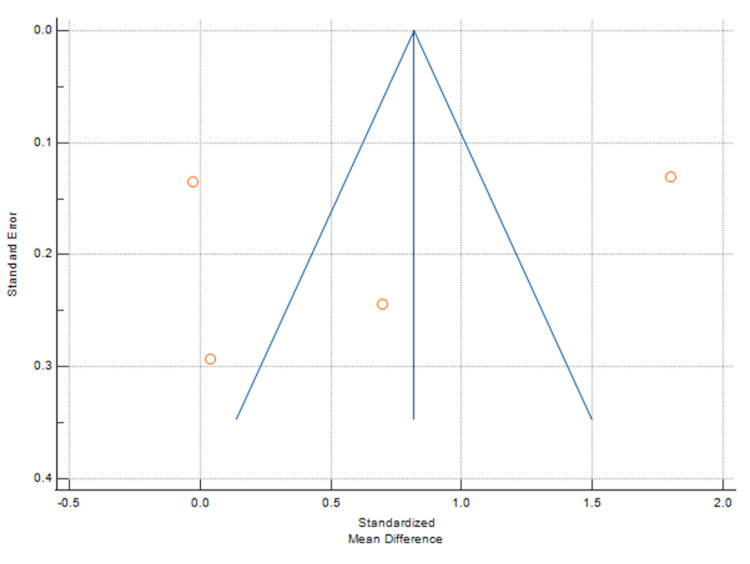
Funnel plot showing change in eGFR. eGFR: Estimated glomerular filtration rate.

Figures 6-7 show the corresponding Forest plot and Funnel plot showing the change in eGFR. 

Risk of bias

The risk of bias was assessed using the Revised Cochrane risk-of-bias tool for randomized trials (RoB 2)[https://methods.cochrane.org/bias/resources/rob-2-revised-cochrane-risk-bias-tool-randomized-trials] for 10 of the 11 studies which were randomized trials. Unfortunately, due to the lack of availability of the full text of the Chinese article by Cao BQ et al. [11], in addition to the language barrier, the risk of bias was not performed for the article. Out of nine articles analyzed, seven studies showed 'some concerns' in the domain of (1) Risk of bias arising from the randomization process. All nine studies were assessed to have a 'low risk' of bias in the domains of (2) Risk of bias due to deviations from the intended interventions (effect of assignment to intervention), (3) Risk of bias due to missing outcome data, (4) Risk of bias in the measurement of the outcome, (5) Risk of bias in the selection of the reported result.

**Figure 8:**
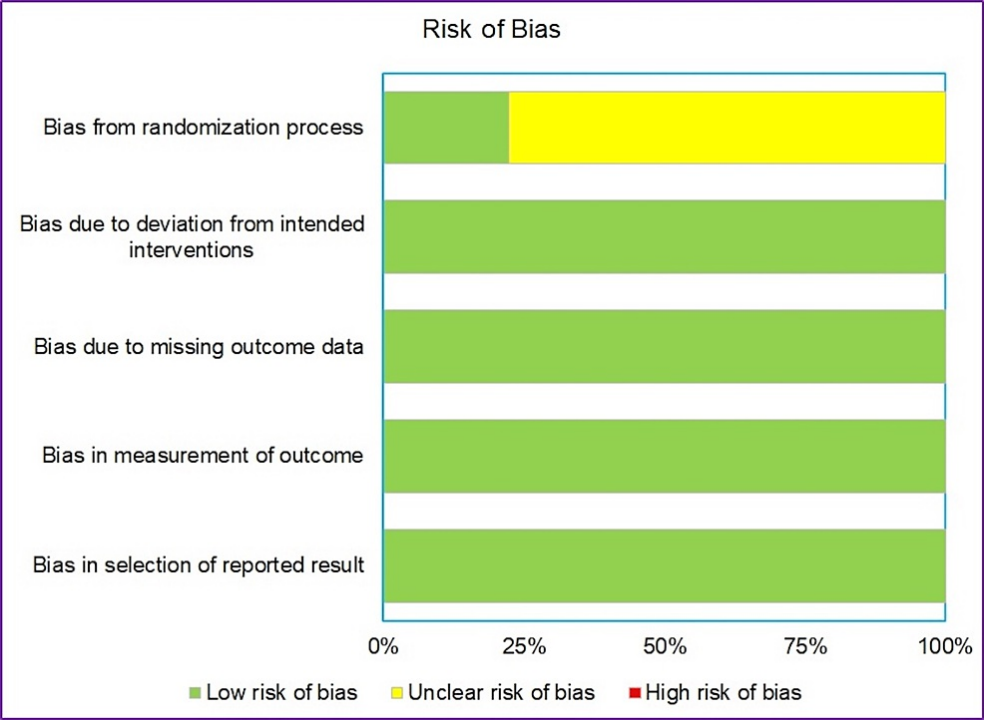
Risk of Bias (Cochrane RoB 2.0 tool). The green bars indicate a low risk of bias, yellow bars indicate an unclear risk of bias (predominantly due to inadequate information regarding the concealment method), and red bars indicate a high risk of bias.

Figure 8 shows the graphical representation of risk of bias analysis. There were no studies that had a high risk of bias for any of the domains.

Discussion

We included 10 RCTs and one retrospective study of high quality in the present investigation to compare the effects of L/N-type CCBs and L-type CCBs on renal functions. The results of our analysis showed that while there were no significant differences in SCr changes between the cilnidipine group and L-type CCB group, the UPE and UPCR were decreased in the cilnidipine group when compared with the L-type CCB group and an increase in eGFR with cilnidipine.

Both UPE and UPCR are indicative of urinary protein quantitative diagnosis. Higher values suggest an immense protein leak from the renal vessel wall compromising renal function. The leaked proteins destroy the Sertoli cells in the glomerulus, resulting in glomerular sclerosis (GS). Urinary protein is closely related to not only chronic renal failure but also independently expedites renal damage and has a negative impact on the cardiovascular system. Therefore, in addition to blood pressure control, proteinuria control is critical in treating hypertension. Cilnidipine appears to be effective in reducing proteinuria or preventing its progression [16,17]. Increased SCr or decreased eGFR suggests a damaged renal function and a risk of ESRD [18,19].

Compared with the L-type CCBs, the L/N-type CCB cilnidipine could significantly lower the UPE and UPCR without increasing SCr or decreasing eGFR, indicating that it was advantageous in improving kidney function in addition to its role in the treatment of hypertension [20]. Furthermore, blood pressure control has been shown to significantly reduce proteinuria [21]. All the 11 trials measured the systolic and diastolic blood pressure in both cilnidipine and L-type CCB groups before and after treatment. The results showed that cilnidipine and L-type CCBs equally decreased blood pressure. Therefore, it was indicated that the reduction in proteinuria in the case of cilnidipine was not caused simply by a fall in blood pressure. Cilnidipine achieved such unique effects through blocking N-type calcium channels, thus inhibiting sympathetic and inducing the dilation of both the afferent and efferent arterioles. Although all the trials had different dose regimens, cilnidipine satisfactorily controlled blood pressure and had a consistent effect on renal functions.

**Figure 9:**
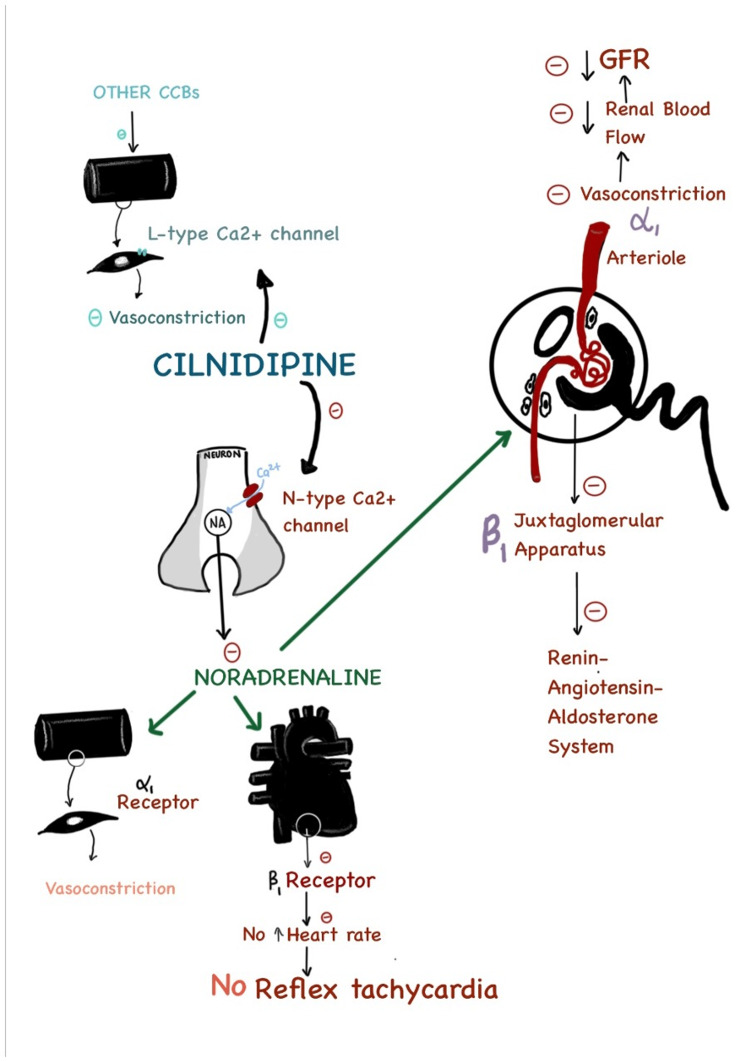
Mechanism of action of cilnidipine. The image has been reproduced by the author (Mayakalyani Srivathsan) of this study, with permission from the source article [22].

Figure 9 shows the unique mechanism of action of cilnidipine. 

Additionally, when analyzed, there was no evidence of significant publication bias.

Our study had its own set of limitations. As it is an observational study, it cannot replace large-scale, multi-centered randomized trials but merely summarise and analyze the existing data. Furthermore, as the trials included were open-labeled, there is a possibility of biases. We considered the sample size in the trials to be quite small. As patient enrolment standards varied from trial to trial, there was heterogeneity among the data. This meta-analysis included only a few studies and more RCTs on a larger scale with long-term follow-up are desirable to confirm these findings further.

## Conclusions

We found that cilnidipine was more effective in reducing proteinuria or preventing its progression and had similar effects on SCr and eGFR in hypertensive patients in comparison to L-type CCBs.
